# A Novel Just-in-Time Intervention for Promoting Safer Drinking Among College Students: App Testing Across 2 Independent Pre-Post Trials

**DOI:** 10.2196/69873

**Published:** 2025-04-10

**Authors:** Philip I Chow, Jessica Smith, Ravjot Saini, Christina Frederick, Connie Clark, Maxwell Ritterband, Jennifer P Halbert, Kathryn Cheney, Katharine E Daniel, Karen S Ingersoll

**Affiliations:** 1 School of Medicine, University of Virginia, 560 Ray C Hunt Dr, Charlottesville, VA, 22903, United States, 1 434-924-8082; 2 University of Virginia, Charlottesville, VA, United States

**Keywords:** alcohol, college students, smartphone intervention, binge drinking, safe drinking

## Abstract

**Background:**

Binge drinking, which is linked to various immediate and long-term negative outcomes, is highly prevalent among US college students. Behavioral interventions delivered via mobile phones have a strong potential to help decrease the hazardous effects of binge drinking by promoting safer drinking behaviors.

**Objective:**

This study aims to evaluate the preliminary efficacy of bhoos, a novel smartphone app designed to promote safer drinking behaviors among US college students. The app offers on-demand educational content about safer alcohol use, provides dynamic feedback as users log their alcohol consumption, and includes an interactive drink tracker that estimates blood alcohol content in real time.

**Methods:**

The bhoos app was tested in 2 independent pre-post studies each lasting 4 weeks, among US college students aged 18‐35 years. The primary outcome in both trials was students’ self-reported confidence in using protective behavioral strategies related to drinking, with self-reported frequency of alcohol consumption over the past month examined as a secondary outcome.

**Results:**

In study 1, bhoos was associated with increased confidence in using protective behavioral strategies. Students also endorsed the high usability of the app and reported acceptable levels of engagement. Study 2 replicated findings of increased confidence in using protective behavioral strategies, and demonstrated a reduction in the self-reported frequency of alcohol consumption.

**Conclusions:**

Bhoos is a personalized, accessible, and highly scalable digital intervention with a strong potential to effectively address alcohol-related behaviors on college campuses.

## Introduction

### Overview

Binge drinking, defined as consuming more than 5 standard drinks for men or 4 for women within a 2-hour period [1,2], is highly prevalent among US college students. This is concerning because binge drinking is linked to various immediate and long-term negative outcomes, including lower academic performance, a higher incidence of sexual assault, drunk driving, motor vehicle accidents, organ damage, and premature death [2-5]. Additionally, research shows that college students consume more alcohol than their noncollege-attending peers [6,7], which is troubling given that excessive drinking during late adolescence and early adulthood is a strong predictor of alcohol use disorders later in life [8]. Therefore, taking proactive steps to address binge drinking among college students is critical to reducing both the short- and long-term consequences in this population.

### Alcohol Interventions in US Colleges

A common approach adopted by US colleges and universities to address hazardous drinking among students involves programs that focus on changing attitudes, increasing knowledge, and modifying behaviors related to alcohol use. Programs like the Brief Alcohol Screening and Intervention for College Students [9,10] typically include brief motivational counseling sessions aimed at reducing students’ positive alcohol expectancies, increasing their awareness of the consequences of drinking, and enhancing their readiness to change [11-15]. Despite the widespread implementation of such alcohol education and intervention programs, national data show that binge drinking rates among college students have remained relatively stable for over a decade [5], highlighting the ongoing difficulty in effectively addressing this public health issue.

### Delivering Alcohol Interventions Through Smartphone Apps

Safer drinking interventions delivered through smartphones may offer distinct advantages in addressing binge drinking among young adults due to their ubiquity, high use, constant presence, and engaging features. Among all US demographic groups, college students have some of the highest rates of smartphone ownership and use [16]. Capitalizing on this widespread adoption provides an opportunity to develop personalized interventions that can effectively engage students and help regulate their alcohol consumption. Compared with in-person interventions, smartphone app-based alcohol interventions are highly accessible, cost-effective, scalable for large student populations, and can offer personalized feedback based on users’ behaviors [17-19].

Researchers have begun exploring the use of smartphone apps to deliver alcohol interventions both in the general population [18-20] and among college students specifically [21-25]. Reviews generally support the feasibility and potential of app-based interventions for addressing outcomes of problematic drinking in college students, though results have been inconsistent across studies [21,23]. One potential reason for this inconsistency may be the lack of sufficient tailoring or personalization for US college students, given that incorporating strategies like gamification to personalize digital interventions may increase user engagement and improve outcomes [26,27]. For example, providing users with dynamic feedback about their drinking, such as their estimated blood alcohol level, may help them to better regulate their alcohol consumption during a night out with their friends to avoid negative consequences. Furthermore, providing users with graphical displays of their current and past alcohol consumption patterns may help them understand how their drinking has impacted other aspects of their lives, such as their academic performance and personal health.

Our team conducted a formative study to gain a detailed understanding of US students’ preferences for interventions and their patterns of smartphone use [28]. Based on our findings and through conducting iterative user-centered design testing, our team developed bhoos (see below for a detailed description of the app and its functions). Similar to other app interventions, bhoos provides on-demand psychoeducational content about safer alcohol use. A novel feature of bhoos, however, is its just-in-time approach. Through an interactive drink tracking feature, students can log their consumption of alcohol in real time, enabling the app to provide dynamic feedback on recommended safe drinking behaviors based on estimated real-time blood alcohol content [28]. The primary goal of bhoos is therefore to promote safer drinking behaviors, in large part by increasing students’ confidence in engaging in protective strategies, with the potential secondary outcome of reducing overall alcohol consumption.

### The Current Studies

We investigated the feasibility and preliminary impact of bhoos in 2 pre-post studies conducted among students at a mid-Atlantic university in the US over a 4-week period. The hypothesis for study 1 was that bhoos would lead to an increase in students’ confidence in using protective behavioral strategies (ie, behaviors that are used while drinking to reduce alcohol use or limit alcohol-related problems) from baseline to postintervention. A secondary hypothesis was that bhoos would lead to a reduction in self-reported alcohol consumption from baseline to postintervention. Study 1 also evaluated the impact of small monetary incentives on drinking outcomes and how students use the app to log their drinks. As study 1 was the first to test bhoos in college students, we examined students’ engagement with the app and their ratings of its usability. Focus groups were conducted at the end of study 1 to gather feedback on the app from participants. Study 2 was conducted as a replication of self-reported drinking outcomes in study 1 to determine the potential of testing bhoos in a future randomized trial.

## Ethical Considerations

Both studies reported in the manuscript were approved by the University of Virginia Institutional Review Board for Social and Behavioral Sciences (IRB-SBS #4020, 5334). All participants provided informed consent. Data are deidentified. Participants in study 1 received gift cards via the web as compensation for completing the baseline (US $20) and postassessments (US $45). Participants randomized to receive added incentives in study 1 received an additional sum, up to US $30, based on their completion of 3 engagement milestones: US $10 for downloading the app, US $10 for logging 1 drink or dry day, and US $10 for logging a “streak” of 3 consecutive days of drinks or dry days. Participants in study 2 received gift cards via the web as compensation for completing the baseline (US $20) and postassessments (US $25).

## Study 1

### Methods

#### Study Overview and Participants

Study 1 consisted of 3 phases. The first phase evaluated the bhoos app in a simple pre-post trial design. The second phase randomized (1:1) participants to receive small additional monetary incentives or not for using the bhoos app in a second pre-post trial. The third phase consisted of focus groups to capture impressions of the app by a subset of participants from the second phase.

For all phases of study 1, college students were eligible to participate if they met the following criteria: (1) aged 18 to 35 years, (2) current enrollment verified via a university email address, and (3) currently owned and used a smartphone. Students were enrolled using traditional, online, and social media methods. Recruitment spanned June through September 2021. Recruitment materials instructed applicants to complete a web-based interest form including contact information, demographic information, and questions to determine initial study eligibility. Research coordinators verified student status and identity with the university’s internal people search. Eligible verified applicants were invited to participate, signed a web-based consent form, and were enrolled in the study.

The phase 1 group included 83 participants (mean age 20.8 years, SD 1.6 years; 68% self-identified as female; 58% self-identified as White, 16% self-identified as Asian or Hawaiian/Pacific Islander, 4% self-identified as Black, 12% self-identified as multiracial, 4% prefer not to answer, and 6% chose to leave the item blank), and phase 2 group included 172 participants (mean age 20.1 years, SD 1.8 years; 60% self-identified as female; 48% self-identified as White, 19% Asian or Hawaiian/Pacific Islander, 8% Black, 11% multiracial, 1% prefer not to answer, and 13% chose to leave the item blank). Assessments occurred at baseline and postintervention, 28 days later. All participants received gift cards via the web as compensation for completing the baseline (US $20) and postassessments (US $45). Participants in the phase 2 group who were randomized to not receive added incentives (n=86) were only provided compensation for completing the baseline and postassessments. However, those randomized to receive added incentives (n=86) received an additional sum, up to US $30, based on their completion of 3 engagement milestones: US $10 for downloading the app, US $10 for logging 1 drink or dry day, and US $10 for logging a “streak” of 3 consecutive days of drinks or dry days (see bhoos description, below).

To improve the app for future trials, the third phase of the trial included a subset of participants (n=18) from the phase 2 group who were invited to be part of debriefing focus groups that occurred 9‐12 months after the initial pre-post trial. A total of 5 focus groups were conducted and qualitative information was collected regarding participants’ general impressions of the app, what features of the app they liked most/least, and the degree to which the app helped them manage their drinking.

#### Intervention: Bhoos

Bhoos (pronounced [booz]) is an app whose name is a play on the word “booze,” slang for alcohol, and the nickname for University of Virginia students, “hoos.” The app provides on-demand educational content about safer alcohol use and dynamic feedback to users as they log their alcohol consumption in real time. Users can log the type of alcoholic drink they are consuming and each drink entry is time-stamped. The app provides real-time estimates of the user’s blood alcohol concentration (estimated blood alcohol concentration) based on their self-identified sex, weight, and number and type of alcoholic drinks logged. In-app notifications are pushed to users based on their estimated blood alcohol concentrations, including information about recommended actions to stay safe and avoid overdrinking. Users can view their current and past drink history through a built-in dashboard. To encourage engagement with the app, users can establish a streak of logins whenever they log consecutive drinking days or dry days (ie, days in which users logged no alcoholic drinks). To encourage engagement, the app also allows users to rate activity level or sleep quality as a secondary health behavior. Materials about safe drinking tips and staying healthy can be accessed directly from the main page. Users can also learn about health-related resources in and around the university through the app on demand. The app design and content were informed by formative research and a think-aloud process with the target population prior to this trial [28].

#### Measures

##### Baseline Alcohol Use Severity

The 10-item Alcohol Use Disorders Identification Test (AUDIT) is the most widely used self-report measure of unhealthy alcohol use [29]. Scores range from 0 to 40, with higher scores indicating more unhealthy alcohol use. Prior studies on college students have suggested cutoff scores for low-risk drinkers (<7), hazardous drinkers (8-15), and alcohol-dependent drinkers (>15). Participants completed the AUDIT at baseline (Cronbach *α*=0.78) to characterize their alcohol use over the past year.

##### Alcohol Consumption in the Past Month

We used a modified 3-item measure based on the Daily Drinking Questionnaire [30] to assess students’ self-reported drinking behavior. Students were initially asked “How often did you drink during the last month?” with response options ranging from 1 (did not drink at all) to 7 (once a day or more). Responses were analyzed as a single-item measure of the average number of days per week in the last month involving alcohol consumption, with higher scores indicating more days per week that involved alcohol consumption. Students who responded that they had drank at least once a month (ie, a score of ≥2) were then asked 2 follow-up questions: “Think of a typical weekend evening (Friday or Saturday) during the last month. How many standard drinks did you drink on that evening?” and “Think of the occasion (any day of the week) you drank the most during the last month. How many standard drinks did you drink?” Students responded to each of these questions by typing a number into an open field. Because these questions were intended to assess for average and maximum alcohol consumption, respectively, we analyzed responses to them as separate items.

##### Protective Behavioral Strategies

A modified version of the 20-item Protective Behavioral Strategies Scale [31-33] was administered at baseline (*α*=0.92) and postintervention (*α*=0.93) to assess students’ confidence in using protective behavioral strategies, or behaviors adopted while drinking to limit their alcohol consumption or limit alcohol-related problems. Specifically, the instructions were modified from the original version to ask students to select the response option that “best fits your confidence level” for using various protective strategies when drinking. Participants responded to each item on a modified scale from 1 (extremely not confident) to 7 (extremely confident), with scores ranging from 20 to 140 [34,35]. Higher scores indicate more confidence in using protective behavioral strategies such as stopping or limiting alcohol consumption, changing the speed or frequency of drinking, and reducing the risk of serious harm by using a designated driver, going home with a friend, or protecting one’s drink from adulterants.

##### App Usability

The 10-item System Usability Scale (SUS) [36] was administered postintervention to assess the usability of the bhoos app. The SUS provides a global view of subjective assessments of usability. We customized the items to specifically mention bhoos. Sample items include “I think that I would like to use bhoos frequently*”* and *“*I thought bhoos was easy to use.” Responses to each item range from 1 (strongly disagree) to 5 (strongly agree). Possible scores on the SUS range from 0 to 100, with a higher score indicating higher overall usability of a system or program. The SUS has been used in roughly 3500 surveys within 273 studies evaluating a range of systems, interfaces, and programs [37]. Internal consistency of the SUS was good (*α*=0.84).

##### Plan for Analyses

To determine whether students’ self-reported drinking outcomes differed across the 2 phases, including the incentive conditions in phase 2, 1-way ANOVAs were conducted using the phase 1, phase 2 incentivized, and phase 2 nonincentivized groups as within participants variables and difference scores for the drinking outcomes as the dependent variables. Difference scores in drinking outcomes between the baseline and postintervention time points served as the dependent variables. Next, for each of the phase 1 and phase 2 groups, paired *t* tests were conducted to obtain estimates of short-term changes in drinking and attitudinal measures from baseline to follow-up. Box-Cox transformations were performed for the drinking outcomes where there was evidence of nonnormality of data based on skewness (outside of −1 and +1) or kurtosis (outside of −2 and +2) being outside of the conventional acceptable ranges. The pattern of findings did not change when using transformed or raw variables. Analyses of self-reported drinking outcomes were performed using IBM SPSS Statistics (version 26.0.0). App use is described using descriptive statistics. Qualitative feedback from focus groups was reviewed by members of the study team (KI, CF, NA, and CC), and recurring themes were identified.

### Results

#### Baseline Alcohol Use Severity

To characterize the sample, we examined AUDIT scores for all participants in study 1. Of the 234 students who completed at least the baseline measures, 133 (59%) were classified as low-risk drinkers (AUDIT Score ≤7), 93 (40%) were classified as hazardous drinkers (AUDIT Score between 8 and 15), and 8 (3%) were classified as alcohol dependent drinkers (AUDIT score >15).

#### Evaluating the Impact of Study Phases on Self-Reported Outcomes

There was no significant effect of the study phase or incentives on any of the self-reported drinking outcomes, for the average number of days per week in the last month involving alcohol consumption (*F*_2, 232_=0.294, *P*=.75, η^2^=.003), typical weekend evening drink consumption in the last month (*F*_2, 165_=0.662, *P*=.52, η^2^=.008), the maximum number of drinks consumed in the last month (*F*_2, 175_=0.005, *P*=.99, η^2^=.00), or protective behavioral strategies (*F*_2, 232_=1.469, *P*=.23, η^2^=.013). Table 1 contains all results of self-reported drinking outcomes, separately for each phase and incentive condition. Below, we report self-reported drinking-related outcomes overall for those in phases 1 and 2 combined, followed separately for each phase and incentive group.

**Table 1.** Self-reported drinking outcomes for study 1.

	Average number of days per week drinking in the last month, mean (SD)				Average number drinks per weekend in the last month, mean (SD)				Max number of drinks on one occasion in the last month, mean (SD)				Protective behaviors, mean (SD)			
	Pre	Post	*t* test (*df*)	*P* value	Pre	Post	*t* test (*df*)	*P* value	Pre	Post	*t* test (*df*)	*P* value	Pre	Post	*t* test (*df*)	*P* value
Phase 1	3.66 (1.31)	3.71 (1.34)	0.54 (75)	.59	3.64 (1.68)	3.61 (1.75)	0.11 (60)	.91	5.68 (3.16)	5.94 (3.12)	0.79 (65)	.43	98.20 (21.01)	100.30 (20.18)	0.98 (75)	.33
Phase 2 (no incentive)	2.99 (1.35)	3.01 (1.36)	0.22 (74)	.83	3.21 (2.06)	2.89 (1.95)	1.35 (50)	.18	4.53 (3.36)	4.59 (3.65)	0.27 (53)	.81	106.19 (24.74)	111.91 (23.28)	2.29 (76)	.03
Phase 2 (incentive)	3.05 (1.52)	2.99 (1.27)	0.55 (81)	.59	3.76 (1.92)	3.93 (2.75)	0.12 (52)	.91	5.93 (3.05)	5.98 (4.48)	0.64 (54)	.52	106.14 (26.05)	108.52 (24.56)	0.89 (80)	.38

#### Protective Behavioral Strategies

Overall, there was a significant increase in students’ confidence in using protective behavioral strategies in drinking from baseline (Mean_pre_ 103.58, SD 24.26) to postintervention (Mean_post_ 106.97, SD 23.20; *t*_233_=2.393, *P*=.02), such that students reported more confidence engaging in more protective behaviors while drinking to limit alcohol-related problems from drinking from baseline to postintervention.

As seen in Table 1, despite an increase in students’ self-reported confidence in using protective behavioral strategies from baseline to postintervention for all 3 groups, the effect was not significant for students in the phase 1 group (*t*_75_=0.98, *P*=.33), and the phase 2 incentive group (*t*_80_=0.89, *P*=.38). There was, however, a significant increase in confidence in using protective behavioral strategies for the phase 2 no incentive group (*t*_76_=2.29, *P*=.03).

#### Alcohol Consumption in the Past Month

Overall, there was no significant change to the average number of days per week in the last month involving alcohol consumption, from baseline (Mean_pre_ 3.23, SD 1.43) to postintervention (Mean_post_ 3.25, SD 1.39*; t*_232_=0.07, *P*=.95). There were also no significant changes to students’ average number of drinks per weekend in the last month from baseline (Mean_pre_ 3.52, SD 1.90) to postintervention (Mean_post_ 3.44, SD 2.20; *t*_164_=0.85, *P*=.40), nor were there significant changes to students’ maximum number of drinks consumed in the last month from baseline (Mean_pre_ 5.43, SD 3.46) to postintervention (Mean_post_ 5.51, SD 3.83; *t*_173_=0.20, *P*=.84).

As seen in Table 1, there were no significant changes to students’ self-reported drinking frequency in the past month from baseline to postintervention for students in the phase 1 group (drinking frequency in the past month, *t*_75_=0.54, *P*=.59; the average number of drinks per weekend in the last month, *t*_60_=0.11, *P*=.91; the maximum number of drinks consumed in the last month, *t*_65_=0.79, *P*=.43). This was also the case for the phase 2 no incentive group (drinking frequency in the past month, *t*_74_=0.22, *P*=.83; the average number of drinks per weekend in the last month, *t*_50_=1.35, *P*=.18; the maximum amount of drinks consumed in the last month, *t*_53_=.27, *P*=.81), and the phase 2 incentive group (drinking frequency in the past month, *t*_81_=0.55, *P*=.59; average number of drinks per weekend in the last month, *t*_52_=0.12, *P*=.91; the maximum amount of drinks consumed in the last month, *t*_54_=0.64, *P*=.52).

#### Usability of Bhoos

There was no significant effect of the study phase or incentives on self-reported usability of bhoos (*F*_2, 234_=0.097, *P*=.908, η^2^=.001). Usability scores for students in phase 1 ranged from 22.5 to 100 (mean 71.22, SD 17.21). Scores for students in the phase 2 nonincentive group ranged from 35 to 100 (mean 71.75, SD 16.96), and 37.50 to 100 (mean 72.38, SD 15.69) for those in the phase 2 incentive group. The average usability score (mean 71.80, SD 16.54) placed bhoos in the third quartile of all programs evaluated by the SUS [37].

#### Engagement With Bhoos

Nearly all participants (93.33%, 238 of 255) in study 1 downloaded the app. The majority of participants (85.49%, 218 of 255) logged at least 1 drink or dry day. Roughly two-thirds (160 of 255) of the participants logged at least 1 alcoholic drink, whereas nearly 3-quarters (189 of 255) logged at least 1 dry day. Slightly more than half (145 of 255) logged at least 1 streak. On average, participants used the app for 12.67 days (SD 9.96 d) of the 28 days of the intervention period.

Overall, providing added incentives did not seem to impact how students used the app to log their drinks. Among those in the phase 2 group, students randomized to receive incentives logged, on average, 11.16 drinks (SD 8.92) while those randomized to receive no added incentives logged, on average, 13.04 drinks (SD 16.62), *t*_130_=0.924, and *P=*.357. Moreover, students randomized to receive incentives logged, on average, 15.50 dry days (SD 9.39) while those randomized to receive no added incentives logged, on average, 15.90 dry days (SD 8.81), *t*_169_=0.282, and *P=*.78.

#### Qualitative Feedback From Focus Groups

The feedback obtained from focus groups revealed several common themes. Students found the bhoos app to be visually appealing and user-friendly. They expressed that the drinking chart within the app raised their awareness of their drinking habits over time, and they appreciated the ability to review their drinking history within the app. However, some students felt that the psychoeducational content within the app was too static, and they suggested that incorporating videos would be beneficial. Additionally, many students expressed a desire to track a broader range of secondary behaviors, including mood and stress.

In response to this feedback from the focus groups, we made several iterative improvements to the bhoos app in preparation for the next pilot study (study 2). Specifically, we enhanced the psychoeducational content by adding 4 short videos on relevant topics, including information on helping an intoxicated friend, knowing the signs of alcohol overdose, learning about alcohol tolerance, and understanding standard drink amounts. We also included more information on safety tips while drinking. We refined the app’s functionality for users to track their mood or stress to identify patterns related to drinking events. Last, known bugs and glitches that students reported while using the app were addressed, enhancing bhoos’ overall performance and reliability.

### Discussion

Collectively, the results from study 1 provide preliminary support for the feasibility and usability of bhoos among college students in the United States. While the students in this sample did not report a reduction in drinking frequency, findings indicate that they were more confident in their ability to engage in protective behaviors while drinking, potentially enhancing their safety. Both app engagement data and mixed methods results confirm the usability of bhoos, while providing added monetary incentives did not seem to impact any of the observed outcomes or how students used bhoos to track their drinking. The qualitative feedback from students was instrumental in identifying areas for app improvement to enhance usability and engagement. Building on these findings, study 2 was conducted to examine whether the impact of bhoos on drinking-related outcomes would be replicated in an independent sample of college students.

## Study 2

### Methods

#### Study Overview and Participants

Eligibility criteria were identical to study 1. College students were recruited over the 2022 Fall semester using advertisements in dining halls. Although the initial goal was to enroll up to 200 students, the recruitment timeline was hampered by the continuation of the COVID-19 pandemic and a tragic event that occurred on university grounds on November 13th, 2022 (a shooting on University grounds that resulted in the murders of 3 students). The latest baseline questionnaires were completed before the event and enrollment of new participants was halted after the event. Participants who completed baseline questionnaires were permitted to complete the study and complete the postintervention questionnaires. In total, 43 students were recruited (60% students self-identified as women; 24 students self-identified as White, 12 students self-identified as Asian or Native Hawaiian, 2 students self-identified as Black, and 5 students self-identified as multiracial). Once again, assessments were administered at baseline and postintervention, 28 days later. All participants received gift cards via the web as compensation for completing the baseline (US $20) and postassessments (US $25). An added incentivization group was not included in study 2.

#### Measures and Plan for Analyses

The same self-report measures were administered in study 2 as in study 1. Specifically, baseline alcohol use severity was assessed by the 10-item AUDIT [29] (*α*=0.82). Alcohol consumption in the past month was assessed at baseline and postintervention with the 3-item measure used in study 1. Each item was treated as a separate single-item scale. The modified 20-item Protective Behavioral Strategies Scale [31-33] was administered at baseline (*α*=0.90) and postintervention (*α*=0.91) to assess students’ confidence in using protective behavioral strategies. Finally, the usability of the bhoos app was assessed postintervention using the SUS [36] (*α*=0.75).

Paired *t* tests were used to investigate short-term changes in drinking and protective behaviors from baseline to follow-up. Due to the limited sample size, if the normality assumption was not met some analyses were computed again with the paired Wilcoxon Signed Rank Test. This did not change the interpretation of any of the results]. Analyses were performed using R (version 4.3.3; R Core Team). App use is described using descriptive statistics.

### Results

#### Baseline Alcohol Use Severity

Of the 43 students, 25 (58%) were classified as low-risk drinkers (AUDIT Score ≤7), 11 (26%) were classified as hazardous drinkers (AUDIT Score between 8 and 15), and 6 (14%) were classified as alcohol dependent drinkers (AUDIT score >15).

#### Protective Behavioral Strategies

As expected, there was a significant increase in students’ confidence in using protective behavioral strategies after using bhoos from baseline (Mean_pre_ 107, SD 17.19) to postintervention (Mean_post_ 113.37, SD 15.87*; t*_31_=-2.79, *P*=.01), such that students reported confidence in engaging in more protective behaviors while drinking to limit alcohol-related problems from drinking after the intervention compared with before.

#### Alcohol Consumption in the Past Month

There was a significant decrease in the average number of days per week drinking alcohol in the last month from baseline (Mean_pre_ 2.32, SD 1.27) to postintervention (Mean_post_ 2.06, SD 1.15; *t*_33_=2.32, *P*=.031). Among students who reported drinking, there was no significant change to their typical weekend evening drink consumption from baseline (Mean_pre_ 3.83, SD 2.80) to postintervention (Mean_post_ 3.87, SD 2.30; *t*_29_=0, *P*=.92), nor was there a significant change to the maximum number of drinks they consumed in a single occasion the last month, from baseline (Mean_pre_ 5.17, SD 4.07) to postintervention (Mean_post_ 5.34, SD 4.50; *t*_28_=0.24, *P*=.82).

#### Usability of Bhoos

Usability scores ranged from 55 to 98 (out of a possible 100). Overall, students reported higher usability of the revised version of bhoos (mean 77.93, SD 11.92) than in the original version tested in study 1. The average score placed bhoos in the upper 75% (fourth quartile) of all programs evaluated by the SUS [37].

### Discussion

Collectively, the findings from study 2 reproduce the drinking-related results from study 1. Specifically, the findings of study 2 replicate the finding that bhoos is associated with an increase in confidence in using protective behaviors while drinking. It is worth noting that students in study 2 also reported reduced frequency of drinking, which may have resulted from improvements to the app from study 1, which is supported by our finding that students rated the app as more usable in study 2 than in study 1.

## Overall Discussion

### Principal Findings

Developing effective, accessible, and scalable interventions to address excessive drinking among college students is critical due to the significant public health concerns posed by alcohol misuse on college campuses. In 2 studies, we observed promising and consistent results regarding the impact and usability of the bhoos app, which effectively promoted confidence in using protective behaviors related to drinking among US college students.

The results suggest that bhoos*’* features, such as drink tracking and dynamic feedback, help to encourage responsible drinking behaviors, including monitoring alcohol intake, planning for safe transportation after drinking, or seeking help when needed. The ability to provide personalized, real-time feedback increases the relevance of the app to the individual needs and experiences of its users. By offering recommendations based on the user’s drinking patterns and risk factors, bhoos may engage users more effectively than a generic one-size-fits-all program which enhances the likelihood of behavior change. Moreover, this more personalized approach to providing risk feedback aligns with the principles of Motivational Interviewing [38,39] and follows proven strategies for promoting behavior change [40-42]. Smartphone app-delivered interventions like bhoos offer several advantages over traditional approaches, including the ability to deliver actionable feedback at critical moments. Additionally, they provide the potential for widespread accessibility and scalability. College students, in particular, maybe the most receptive to mobile apps for obtaining information and support for health-related decisions, given their high smartphone use [16]. The ease of dissemination and the low cost of smartphone apps make them a practical option for broad implementation on college campuses.

### Limitations and Future Directions

The findings from our studies should be considered in light of several limitations. First, the reliance on infrequent and static measures of drinking behaviors introduces the possibility of recall bias. Moreover, the primary outcomes of both studies reflected students’ confidence in using various protective behaviors while drinking and not the actual behaviors they engaged in. Future research could benefit from incorporating more ecologically valid approaches, such as ecological momentary assessment, perhaps administered within the app, to assess for actual protective behaviors related to drinking while limiting recall bias. Additionally, the pre-post design of our study limits our ability to evaluate the efficacy of bhoos, which could be better assessed through a randomized controlled trial. The short time frame between baseline and postassessment (4 weeks) restricts our understanding of the long-term sustainability of the app’s effects. Future studies should explore the app’s impact over longer periods, such as a full college semester. Moreover, a large percentage of students in both studies were White and female which may limit the generalizability of our findings to minorities and men. Finally, the tragic shooting that occurred on university grounds may have influenced students’ drinking behaviors in study 2, although the extent of this impact is unclear. Future studies should examine how such events, and events more broadly (eg, political elections, performance of University athletic teams), affect student drinking behaviors.

### General Conclusion

In summary, preliminary findings across 2 studies indicate that bhoos shows strong potential as a tool for promoting protective behaviors related to alcohol use among college students. While these studies help advance highly accessible and scalable approaches to address the consequences of binge drinking by US college students, further research is needed to fully understand the app’s role in comprehensive alcohol harm reduction efforts within this population. Its personalized approach, combined with its accessibility and scalability, suggests that bhoos could be a valuable addition to the range of interventions available to address alcohol-related issues on campuses.

## Abbreviations

AUDIT: Alcohol Use Disorders Identification Test
SUS: System Usability Scale
